# Rate of bacteremia in the hemodialysis patient presenting to the emergency department with fever: a retrospective chart review

**DOI:** 10.1186/s12245-018-0188-5

**Published:** 2018-05-25

**Authors:** Nicholas Villalon, Neda Farzan, Kathryn Freeman

**Affiliations:** Department of Emergency Medicine, Commonwealth Healthcare Corporation Hospital, Commonwealth of the Northern Mariana Islands 1 Navy Hill Dr. , Box 10002 PMB 4211, Saipan, MP 96950 USA

**Keywords:** Hemodialysis, Febrile, Fever, Emergency department, Bacteremia, Sepsis

## Abstract

**Background:**

Infectious disease is the second most common cause of death in patients receiving hemodialysis (HD). When presenting to the emergency department (ED) with fever, it remains a diagnostic challenge to distinguish patients with potentially life-threatening bacterial infections from those with less significant causes of fever. The primary goal of this study was to determine the rate of bacteremia in HD patients presenting to the ED with fever. The secondary goal of this study was to identify any independent risk factors associated with bacteremia in the febrile HD patient.

**Methods:**

This is a retrospective medical record review of all HD patients who presented to the ED with either subjective fever as primary complaint or with a documented triage temperature of 38 °C or higher during the 3-year period between September 1, 2014, and September 1, 2017. Patient visits were included in the study if blood cultures were ordered in the ED. Data related to demographic information, clinical parameters, diagnostic test results in the ER, final diagnosis, and results of microbiology cultures were collected from each patient encounter. Univariate analysis was performed to identify risk factors associated with bacteremia.

**Results:**

We identified 353 patient visits from 138 unique patients that met inclusion criteria. Fifty-eight percent of these were women, and the average age was 54.6 years. The rate of bacteremia was 31.7%, and the main microorganisms isolated in blood culture were non-MRSA *Staphylococcus aureus* (40.7%), MRSA (13.3%), Pseudomonas aeruginosa (11.5%), and Enterobacter spp. (11.5%). Independent prognostic factors associated with bacteremia were use of dialysis catheter, prior history of bacteremia, and > 5% neutrophilic band cells (OR 6.55 [95% CI 3.96–10.8; *p* < 0.0001]; OR 8.87 [95% CI 5.32–14.8; *p* < 0.0001]; OR 3.32 [95% CI 1.90–5.80; *p* < 0.0001] respectively).

**Conclusion:**

HD patients presenting to the ED with fever have high rates of bacteremia, with a significantly higher rate in patients using dialysis catheters or those with a history of bacteremia. Other clinical data available in the ED is minimally useful in predicting bacteremia.

## Background

Infection is the second most common cause of death in patients dependent on hemodialysis (HD) [1–4]. Fever is a common presenting symptom of infectious disease, and differentiating HD patients with simple febrile illness from those with potentially life-threatening bacterial infections continues to be a diagnostic challenge [5]. HD patients visit the emergency department (ED) about six times more often than their age-matched counterparts, and the second most common reason for admission to the hospital is septicemia [6, 7].

Micronesians have particularly high rates of hypertension, diabetes, and renal disease compared to other Asian and Caucasian ethnic groups, and a disproportionate burden of kidney failure requiring hemodialysis [8, 9]. The ED in our hospital sees a relatively high number of HD patients presenting with infectious symptoms.

While the burden of initially identifying patients with septicemia often falls on the ED provider, the studies that have been done to evaluate risk factors for potentially life-threatening bacterial disease have been largely retrospective studies from already-confirmed cases of bacteremia [10–16].

The aim of this study was to determine the rate of bacteremia in HD the patient presenting to the ED with fever and to identify independent risk factors for bacteremia in this cohort.

## Methods

### Study design

Commonwealth Healthcare Corporation Hospital (CHCC) is the only hospital serving the island of Saipan, a US commonwealth with a population of about 50,000. The emergency department sees an average yearly volume of approximately 18,000 patient visits. We conducted a retrospective chart review in a cohort of dialysis-dependent patients presenting to the ED with fever from the 3-year period between September 2014 and September 2017.

### Eligibility criteria

Eligible subjects were patients with a history of end-stage renal disease (ESRD) dependent on dialysis who presented to the ED with either a complaint of fever or a fever documented upon triage of 38 °C or higher, with blood cultures drawn in the ER. We excluded patients on peritoneal dialysis, patients with no blood cultures drawn, incarcerated patients, and patients less than 18 years of age.

### Data collection

For each included patient, one of the three authors reviewed the medical chart and recorded clinical data in a Microsoft Excel spreadsheet. The reviewers were all active clinical staff in the ED where the patients were enrolled, and familiar with the electronic medical record (Resource and Patient Management System, Electronic Health Record. Rockville, MD.). The data points collected were as follows: date of ED visit, sex, age, race, HD access, HD frequency, comorbidities (diabetes mellitus, hypertension, coronary artery disease), initial vitals in the ED (temperature, heart rate, blood pressure, oxygen saturation by pulse oximetry, respiratory rate), laboratory data (white blood cell count, %neutrophils, %neutrophil bands, C02, urinalysis, rapid flu), radiology data (ED provider interpretation of chest radiograph), microbiology data (results from blood cultures, wound cultures, and urine cultures), and clinical assessment data (ED provider diagnosis, disposition from ED, internal medicine discharge diagnosis, ICU stay, 7-day mortality, and 28-day mortality). A random sampling of 10% of all charts was reviewed by the principal investigator to ensure accuracy and internal consistency between co-investigators.

### Microbiologic evaluation

Blood cultures were collected upon presentation to the ED, and isolates obtained from standard bacterial blood culture automated detection system (BD Bactec™ 9120, Becton Dickinson, Franklin Lakes, NJ, USA) and microbial identification and antibiotic susceptibility testing system (Vitek® 2 Automated ID/AST instrument, bioMérieux, Marcy l’Etoile, France) were identified and reported by clinical microbiology staff at CHCC. Per hospital policy, two aerobic blood culture samples were processed for each patient.

### Study definitions

Bacteremia was defined as bacterial growth detected by blood culture testing that was determined to be an etiologic pathogen (non-contaminant) by clinical hospital staff. Similarly, urinalysis results were defined as positive for urinary tract infection as determined by ED provider interpreting the initial result of the urinalysis. Definition of pneumonia on chest radiograph was defined as an interpretation of pneumonia as determined by ED clinician interpreting the study at the time of the ED visit. Because some patients had more than two types of vascular access (e.g., permanent vascular catheter as fistula or graft was maturing), vascular access was categorized by hierarchy of perceived risk for bacteremia: dialysis catheter > arteriovenous graft (AVG) > native arteriovenous fistula (AVF). A patient was considered to have a “history of bacteremia” for any diagnosis of bacteremia documented in the medical record after initiation of HD, even if this occurred before the 3-year study period.

### Primary outcome

The primary outcome of the study was bacteremia confirmed by microbiologic testing in patients initially presenting to the ED with fever.

### Statistical analysis

Categorical data was presented based on absolute and relative frequencies and numeric variables presented based on their mean and standard deviation where applicable.

For comparison of risk factors associated with bacteremia, non-parametric data were analyzed using Taylor Series risk ratio tests. The level of significance for the test was two-sided, with *α* < 0.05.

## Results

Over the 3-year study period, 269 unique HD patients made a total of 3424 visits to the ED. This corresponded to 2.6 visits/patient or 0.87 visits/patient/year. Of these visits, 358 (10.5%) were for complaint of fever and 353 (10.3%) prompted a septic workup that were ultimately included in the study. Over the study interval, 51.3% of all known HD patients in our community had one or more visits to the ER for fever.

The 353 visits included in the study were made by 138 unique HD-dependent patients. Average patient age was 54.6 years (SD 12.7 years), and more than half of patient visits were made by women (60.6%). The great majority of patients were ethnically Chamorro (66.9%), and the remainder was other Pacific Islander (18.1%) and Filipino (9.6%). 43.3% were being dialyzed using permanent tunneled catheter, 39.1% native AVF, and 17.6% AVG. 67.4% of patients were admitted to the hospital, 31.2% were discharged from the ED, and 1.4% left against medical advice. Total 7-day mortality was 2.8%, and 28-day mortality was 5.4%. In patients with confirmed bacteremia, 7-day mortality was 3.6%, and 28-day mortality 9.8%.

There was a high percentage of comorbidities, with diabetes mellitus (79.0%), hypertension (92.1%), and coronary artery disease (60.6%). Major causes of fever were found to be pneumonia, urinary tract infection, dialysis catheter infection, and skin and soft tissue infection (Table 1).

**Table 1:** Baseline characteristics of hemodialysis patients presenting to the ED with fever

	*n*	(%)
Total	353	100.0
Sex (F)	214	60.6
Age	54.6	12.7
Race		
Chamorro	236	66.9
Carolinian	25	7.1
Filipino	34	9.6
Pohnapean	10	2.8
Palauan	17	4.8
Chuukese	29	8.2
Taiwanese	2	0.6
Disposition		
Admit	238	67.4
Discharge	110	31.2
AMA^a^	5	1.4
Access		
Cath	153	43.3
AVF	138	39.1
AVG	62	17.6
Risk Factors		
HTN	325	92.1
DM	279	79.0
CAD	214	60.6
Source of Fever		
Pulmonary	91	25.8
Urine	63	17.8
HD catheter	61	17.3
SST^b^	59	16.7
Viral	38	10.8
FUO	33	9.3
Abdomen	4	1.1
Bone	4	1.1

^a^Left against medical advice

^b^Skin and soft tissue infection

### Outcome

The rate of bacteremia in HD patients presenting to the ED was 31.7%. Gram-positive organisms accounted for 61% of positive blood cultures, with a predominance of MSSA (36.3%%) followed by MRSA (13.3%). In the Gram-negative bacteremia group, major pathogens included *Enterobacter* (11.5%), *Pseudomonas* (11.5%), *Escherichia coli* (3.5%), and *Klebsiella* (3.5%) species (Table 2).

**Table 2:** Blood culture isolates from confirmed cases of bacteremia

	*n*	(%)
Gram-positive bacteria	69	61.1
*S. aureus*		
nMRSA	41	36.3
MRSA	15	13.3
Other *Staph spp.* ^a^	5	4.4
*Enterococcus spp.* ^b^	4	3.5
*Streptococcus*^c^	3	2.7
*Bacillis spp.*	1	0.9
Gram-negative bacteria	44	38.9
*P. Aeruginosa*	13	11.5
*Enterobacter spp.* ^d^	13	11.5
*E. Coli*	4	3.5
*K. pneumoniae*	4	3.5
*Providencia stuartii*	4	3.5
*Other Gram-negatives*^e^	6	5.3
Total number of isolates	113	

^a^Capitis, auricularis, lungdensis, epidermitis, cohnii

^b^Faecium, faecalis

^c^Group A *Strep*

^d^Cloacae, aerogenes, absuriae

^e^*Strenotrophamonas maltophilia*, *Aeromonas hydrophilia*, *P. Mirabilis*, *C. Koseri*

**Fig. 1:**
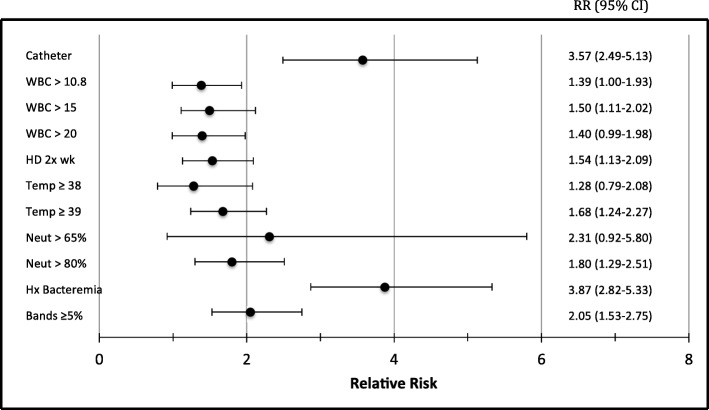
Relative risk for bacteremia

By univariate analysis, the factors most strongly associated with bacteremia were hemodialysis catheter use (RR 3.57 (2.49–5.13)), history of bacteremia (RR 3.87 (2.82–5.33)), and bandemia ≥ 5% (RR 2.05 (1.53–2.75)) (Fig. 1).

## Discussion

Compared to the general population, hemodialysis patients have higher rates of bacteremia and associated mortality [17, 18]. Previous work studying this population has relied largely on confirmed cases of bacteremia, but the authors of this study are not aware of any ED-based studies concerned with the evaluation of the yet undifferentiated HD patient in whom bacteremia is only suspected. The primary goal of this study was to evaluate the rate of bacteremia in the HD patient presenting to the ED with fever, in the hopes of developing a more clinically useful framework for clinicians managing initial presentation in the ED and other outpatient settings.

The incidence of bacteremia in the HD patients in our population was found to be similar to that of previous studies [19, 20]. Compared to other hemodialysis patients in Asia, Europe, and the USA, our patients had a higher percentage of comorbidities (DM, HTN, CAD), a higher rate of dialysis catheter use, and were younger on average [21–25]. Mortality from sepsis in bacteremic patients in our study was similar to previous studies, with 7-day mortality of 3.6% and 28-day mortality of 9.8% [16]. Consistent with other studies, the predominant sources of bacteremia were found to be infections of the hemodialysis catheter, lungs, urinary tract, and skin and soft tissue. Gram-positive organisms accounted for the majority of organisms identified in blood culture (61%)—predominantly *Staphylococcus aureus*—but unlike prior studies, methicillin-sensitive *S. aureus* was more common than MRSA [26–30].

History of bacteremia and presence of a hemodialysis catheter were found to be the most significant risk factors for bacteremia in HD patients presenting with fever. In our study, more than half (53.6%) of the patients with a catheter presenting with a fever were ultimately found to be bacteremic, and the majority of the bacteremic cases were found to be caused by catheter infections. The risk of infection with hemodialysis catheter has been well documented, and this result is consistent with prior studies [31, 32]. For non-catheter HD patients, the rate of bacteremia was significantly lower (15.0%), and pulmonary and skin and soft tissue sources accounted for the majority of cases of bacteremia. While bandemia greater than 5% had some correlation with bacteremia (RR 2.05 (1.53–2.75)), ultimately, the other diagnostics generally available at first presentation (documented temperature, white blood cell count, neutrophilia) were of minimal significant clinical value as risk factors in predicting bacteremia.

This study has several limitations. This is a retrospective study, from a single center, with a study population that may be difficult to generalize to other clinical settings. Also, only aerobic blood cultures were drawn in this study, so bacteremia caused by obligate anaerobes would have been missed in our data set, though these infections are thought to be relatively infrequent [33]. Finally, there was some data not available for all patients, and it is possible that this introduced some bias into our analysis.

## Conclusion

Hemodialysis patients carry a high burden of infectious disease, a high mortality with serious infections, and it remains challenging to predict which patients presenting to the ED with fever are bacteremic. In this study, we found that previous history of bacteremia and presence of a hemodialysis catheter were strong risk factors for the presence of bacteremia. We also found that leukocytosis, documented fever, and neutrophilia offered little predictive value in identifying patients with bacteremia.

## Abbreviations

AVF: Arteriovenous fistula
AVG: Arteriovenous graft
CAD: Coronary artery disease
CHCC: Commonwealth Healthcare Corporation Hospital
DM: Diabetes mellitus
ED: Emergency department
ESRD: End-stage renal disease
HD: Hemodialysis
HTN: Hypertension
MRSA: Methicillin-resistant *Staphylococcus aureus*
MSSA: Methicillin-sensitive *Staphylococcus aureus*
